# Psychological distress and academic success: a two-year study comparing the outcome of two online interventions at a university counseling and consultation service in Italy

**DOI:** 10.3389/fpsyt.2024.1427316

**Published:** 2024-10-08

**Authors:** Silvia Mammarella, Laura Giusti, Sasha Del Vecchio, Anna Salza, Massimo Casacchia, Rita Roncone

**Affiliations:** ^1^ Department of Life, Health and Environmental Sciences, University of L’Aquila, L’Aquila, Abruzzo, Italy; ^2^ University Unit Rehabilitation Treatment, Early Interventions in Mental Health—San Salvatore Hospital, L’Aquila, Italy

**Keywords:** online intervention, cognitive-behavioral therapy, young people, clinical psychology, mental health, synchronous vs asynchronous technologies, academic outcome, Italian university counseling services

## Abstract

**Background:**

The mental health of college students has been a significant concern in higher education, representing a priority for the institutions. Enhancing effective online interventions could represent excellent support for university counseling services that deal with students’ well-being. The present study aimed to evaluate the effectiveness of two online interventions provided by the Counseling and Consultation Service for Students (SACS) of the University of L’Aquila (Italy), namely Videochat with Clinical Mental Health consultation (VCMH) and Therapist-supported Internet Cognitive Behavioral Therapy (Ts-iCBT), a 12-module asynchronous program for anxiety management. The primary outcome was focused on the emotional well-being of students (GHQ-12). The secondary academic outcome was represented by the attainment of their final degree.

**Methods:**

Students requesting help from the SACS between March 2020 and March 2022 during different periods of the COVID-19 pandemic restrictions were asked to fill out a brief structured screening tool and access the PSYDIT.COM platform. Based on their personal preferences, students chose to access one of the two different online interventions: VCMH and Ts-iCBT.

**Results:**

Over the two-year duration of this study, 379 students (267 women, 70.4%) contacted the service. Out of 334 students, 72.5% asked to be included in the Ts-iCBT group, whereas 27.5% expressed a preference for the VCMH group. A statistically significant decrease in emotional distress was obtained by both forms of online intervention, highlighting a better outcome for VCMH. In addition to clinical variables, 28.8% of students who completed online interventions attained their degree, with a statistically significantly higher percentage of graduated students in the Ts-iCBT compared to VCMH group. A high proportion of students (37.7% of total sample) dropped out (DO) of digital interventions, with a statistically significant lower rate of dropouts in the VCMH group.

**Conclusions:**

The effectiveness of online interventions is extremely promising and yields a growing source of suggestions for use in providing more tailored interventions. Videochats and frequent therapist online contacts would be recommended for more severe psychopathological conditions. Students in conditions of moderate and non-severe emotional distress who feel more able to “manage the problem alone” could be addressed to asynchronous online interventions.

## Introduction

University students represent the future of our nations, thus justifying a major focus on their education and mental health. University students are frequently faced with a series of challenges, including demanding studies, moving to new cities, and adapting to a new and competitive academic environment. These challenges may result in homesickness, loss of social support (1), and feelings of loneliness (2), at times heralding the onset of symptoms of depression and anxiety (3–5). A mental health crisis may impact heavily on students’ cognition, emotions, and behavior (6), in addition to prejudicing academic performance (7). Moreover, personal and socio-contextual variables may influence students’ educational pathway and commitment (8–10).

The mental health of university students has long represented a priority concern in the area of higher education (11). University counseling services have focused on the early detection and treatment of emotional distress, identifying the most appropriate facilities and most effective interventions to address the needs of their students (6, 12).

University counseling services constitute a valuable resource in the individual and professional development of students, providing access to coping strategies and skills to help them manage academic and personal difficulties. To date, several studies have demonstrated the effectiveness of face-to-face counseling services, underlining their importance in promoting mental health (13–15), reducing psychological distress (16) and improving academic results (17). Since 1991, the Counselling and Consultation Service for Students (SACS) at University of L’Aquila, Italy has provided a “safe place” for students to express their emotional distress and be guided in their academic courses (18) also during the emergency of April 6, 2009, when a devastating earthquake hit L’Aquila and brought death and destruction to the University of L’Aquila, killing 55 students (19). Interventions available from the SACS include individual and group cognitive-behavioral training for the management of anxiety, with specific focus on the cognitive restructuring of dysfunctional thoughts (20) and computerized interventions (21).

The COVID-19 pandemic, in addition to producing an increase in the prevalence of anxiety, depression, and post-traumatic symptoms among university students (22–24), interrupted face-to-face evaluation and interactions in university counseling, resulting in a substantial acceleration of digital cognitive-behavioral therapy (CBT) approaches on websites and web applications (25). Systematic reviews have found how remote internet-based interventions are as effective as those conducted in-person across a range of psychiatric diagnoses and patient populations, reporting a high user satisfaction (26–28), as well as therapists (29).

Literature reports describe a series of positive experiences and testimonies of digital counseling services provided by Italian universities, underlining the benefits of video chat technology in providing access to expert mental health professionals and prescription of digital mental health interventions (22, 30–33).

Among these examples, the following stands explicitly: the majority of these studies adopted real-time video chats (30–32), representing synchronous technologies to allow the user and therapist to communicate simultaneously, demonstrating its effectiveness in different settings of use.

Among these positive experiences an interesting perspective could be also opened by the structured use of asynchronous technologies (i.e. SMS text messaging, e-mail, narrative diary, computerized guided therapy, mobile app-based psychotherapy, and psychoeducation) as an alternative to traditional synchronous technologies (22–33), allowing users and therapists to communicate without the need for simultaneous communication (34). The Italian group led by Paganin (33) highlighted student acceptance of smartphone-based interventions for stress management and promotion of well-being. Our group evaluated a “cognitive-behavioral computerized guided therapy” which proved as effective as person-to-person CBT in the treatment of adults and young people with anxiety disorders (21); a similar program has been in use in our SACS since March 2020, following the onset of the COVID-19 pandemic in wich the service has been made available to students and young people exclusively via a digital platform (22).

Compared to synchronous technology, delays in therapist responses, patient use of messaging during acute crises, and potential misinterpretations of text communications are potential sources of frustration for users. However, therapists should educate users about the appropriate timing, methods, and use of technologies to reduce miscommunication and consequent frustration (34). This study therefore aimed to evaluate the effectiveness of two online interventions provided by the Counseling and Consultation Service for Students of the University of L’Aquila (Italy) (SACS), namely Videochat with Clinical Mental Health consultation (VCMH) and Therapist-supported Internet Cognitive Behavioral Therapy (Ts-iCBT) a 12-module asynchronous program for the management of anxiety. The primary outcome was students’ emotional well-being. Since the literature outlined that the emotional well-being can impact academic performance and vice versa, the secondary academic outcome in the present study was represented by the degree attainment (35, 36).

## Materials and methods

### Study design

The present study is part of a currently ongoing project which started in March 2020 and was conducted through the digital platform of the SACS of the University of L’Aquila (Italy) (22).

Preliminary data from this project have been published and are referred to the first 2-month period of the Italian lockdown from March 16, 2020 to May 4, 2020 (22). It emerged that out of 103 of our help-seeking students who accessed the SACS service, 21.4% experienced lockdown as a traumatic experience due to adjustment to the new academic activities, lack of autonomy, and conflicts with family members. Furthermore, 36% of our student sample reported suffering from anxiety and depressive symptomatology related to the problematic thinking style “all or nothing”.

This study analyzed a two-year period of intervention ranging from March 2020 to March 2022. All students seeking help from the SACS were asked to access the platform PSYDIT.COM through their institutional mail and register in the protected digital area on receipt of a personal confirmation e-mail.

The platform PSYDIT.COM is a protected digital environment that combines all necessary psychotherapy tools, ensuring full confidentiality of health data as provided for by the European General Data Protection Regulation n. 2016/679. The PSYDIT.COM platform is an IT-telematic system that allows professionals and users to monitor treatment in clinical practice.

PSYDIT.COM facilitates digital communication, transferring it from a random, unprotected, and unmanaged context, such as e-mails or WhatsApp, to a structured and privacy-protected communication and listening pathway.

As a first step, students were asked to fill out a short form to provide key socio-demographic and clinical information, including age, gender, place of residence, off-site student condition, and previous contact with mental health services, including prescription of psychopharmacological treatment. Furthermore, students were asked to complete an assessment screening battery (see below, Assessment Battery).

The second step included a narrative diary. Students were asked to write down the difficulties they were experiencing by responding to the following narrative stimuli, adapted from the narrative-based medicine questions and prompts (37, 38):

1. What are your main worries?
2. How is this situation affecting your life?
3. What kinds of unpleasant emotions are you feeling?
4. What kinds of unpleasant thoughts go through your mind?
5. How can we help you?

Once responses had been provided, the person entered a virtual clinical “room” with professional therapists and used protected messaging and video-chat system to communicate, according to a shared calendar.

Furthermore, students were able to use their digital diary whenever they wanted and share their emotional condition. Narrative data from diaries were not analyzed in the present study.

Clinicians offered an introductory video chat to meet the student and discuss the main issues raised, together with the results of battery scoring. They also explained the main characteristics of the two proposed interventions.

Based on their preferences, students were allocated to either 1) VCMH or 2) asynchronous Ts-iCBT for anxiety management.

Whereas severe symptoms were present, assessed through the screening battery and an initial interview with the assigned therapist, the psychiatrist of the SACS (RR) carried out a diagnostic assessment according to the criteria of DSM-5, as well as integrating psychopharmacological support if necessary.

All participants provided written informed consent to take part in the study.

#### Assessment battery

Subjects in both groups were assessed at the start of the study (T0) by means of the following psychological battery: 12-item General Health Questionnaire (GHQ-12) (39–41); Self-Rating Anxiety Scale (SAS) (42); Beck Depression Inventory-II (BDI-II) (43); Impact of Event Scale-Revised (IES-R) (44). This standardized test battery was chosen because it allows for a broad-spectrum assessment of anxiety and depressive symptoms, the presence of post-traumatic and emotional distress, which were very common in young adults both before and during the pandemic and still are (45, 46).

On completion of interventions (T1), 3 months from T0, students were reassessed. In this study, at T1, GHQ-12 was the only outcome variable considered.

##### Anxiety and depressive symptomatology

###### 12-item general health questionnaire

The GHQ-12 (39–41) is the most extensively-used screening instrument for common mental disorders, in addition to being a more general measure of emotional well-being. GHQ-12 consists of 12 items, each assessing the severity of a mental issue manifested over the past few weeks using a 4-point Likert-type scale (from 0 to 3). A total score ranging from 0 to 36 is generated, with higher scores indicating poorer health. Scores fall into three categories: 0–14 = normal range, 15–19 = moderate psychological distress, and 20–36 = severe psychological distress.

###### Self-rating anxiety scale

The SAS (42) comprises 20 items investigating anxiety symptomatology, including five items investigating well-being (the latter requires reversed scores). Items are evaluated on a 4-point Likert scale (ranging from 1 = “nothing or only for a short time” to 4 = “continuously or most of the time”). Total raw scores range from 20 to 80, with higher scores associated with greater severity of symptoms. Clinical interpretation of the level of anxiety is as follows: 20–44 = normal range, 45–59 = mild to moderate anxiety, 60–74 = marked to severe anxiety, and 75–80 = extreme anxiety.

###### Beck depression inventory-II

The BDI-II (43) is a 21-item inventory measuring the severity of self-reported depression manifested over the previous two weeks; item content corresponds to criteria for the diagnosis of depressive disorders as specified in the Diagnostic and Statistical Manual of Mental Disorders IV, DSMIV. Items are structured on a 4-point scale, ranging from zero (symptom not present) to three points (symptom strongly present). Thus, a BDI-II total score ranging from 0 to 13 points represents normal to minimal depression, from 14 to 19 points mild depression, from 20 to 28 points moderate depression, and from 29 to 63 points indicates severe depression.

###### Traumatic distress

###### Impact of event scale-revised

The IES-R is one of the most widely used self-reporting measures in the field of traumatic stress (44). The IES-R consists of 22 items with a 5-point Likert-type scale ranging from 0 (not at all) to 4 (often). Three subscale scores can be obtained by summing the relevant item scores: intrusion, avoidance, and hyperarousal. Total IES-R score is divided into 0–23 (normal), 24–32 (mild psychological impact), 33–36 (moderate psychological impact), and >37 (severe psychological impact).

#### Online interventions

The SACS includes a multidisciplinary team composed of all CBT-certified psychotherapists, i.e. clinical psychologists, and psychiatrists and psychiatric rehabilitation technicians (47) (the latter are well-trained in CBT psychoeducation strategies). All therapists who agreed to take part in this study created their profiles on the digital platform PSYDIT.COM.

Before starting the study, on November 20th 2019, one of the therapists (AS) went to Rome to the headquarters of the start-up Digital Narrative Medicine (DNM) srl that created the PSIDYT.COM platform to be trained on the technical use of the platform. Subsequently, AS trained the other therapists in February 2020 at the University of L’Aquila on how the platform works, acquiring good skills for accessible functions. This peer support also took place during the lockdown period through online meetings held on Teams.

Four therapists took part in this study (LG, SM, SDV, AS). Therapists attended weekly group supervisory sessions to build upon knowledge obtained on the platform. The dedicated SACS psychiatrist (RR) was available for students who presented with severe psychiatric symptoms at first interview.

##### Video-Chat with Clinical Mental Health consultation, VCMH

VCMH 60-minute individual consultations were conducted on a weekly basis. Consultations included psychoeducation, stress management, cognitive restructuring, problem-solving, and relapse prevention. The role of the therapist was to provide coping strategies for stressful events and emotional suffering, particularly related to academic issues. Therapists guaranteed greater flexibility in structure of the interventions, which also included addressing emerging emotional needs and increased frequency of sessions. The scheduled intervention foresaw the attendance of 12 sessions, although timing of the intervention was flexible to fit with attainment of established student goals.

##### Therapist-supported iCBT for anxiety management, Ts-iCBT – asynchronous program

Students were able to individually access the 12 modules of Ts-iCBT for anxiety management. The contents of the program utilized in the Ts-iCBT group are summarized in **Table 1**. (21) Students were able to enter the next session with a latency of one week between sessions to give them time to study the materials presented and complete the required homework.

**Table 1:** Session contents of Therapist-supported iCBT for anxiety management, Ts-iCBT – asynchronous program (21).

	Session content	Homework assignment
**Sessions 1–2** **Orient the patient to CBT/psychoeducation**	Orient the patient to CBT. • Psychoeducation about the common signs and symptoms of anxiety disorders • Set initial treatment plan/goals	1. Read the user’s manual section on anxiety disorders 2. Monitor the achievement of established weekly goals
**Sessions 3–4** **Anxiety management strategies**	Acquire specific relaxation skills • Explain the rationale for relaxation strategies • Deep breathing • Muscle relaxation	1. Read the user’s manual section on specific relaxation skills 2. Daily diary of deep breathing exercises 3. Daily diary of muscle relaxation exercises
**Sessions 5–8 Cognitive therapy/thinking strategies**	Introducing the cognitive model • Explain the rationale for examining thinking patterns • Review the relationship between thoughts, feelings, and behaviors • Explain the ABC model (activating event, beliefs, emotional and behavioral consequences) • Identifying maladaptive thoughts and beliefs • Focus on ‘jumping to conclusions’ bias • Bias against disconfirmatory evidence, BADE • Suggest or generate alternative, more functional thoughts/beliefs • Challenge of self-injurious thoughts and feelings through Cognitive Restructuring form	1. Read the user’s manual section on specific problematic thinking styles 2. Daily diary of unpleasant situations 3. Daily diary of maladaptive thoughts and beliefs 4. Practice with the cognitive restructuring module
**Sessions 9–11 Structured problem solving**	Introduce rationale and when to problem-solve • Explain the steps to effective structured problem - solving and practice	1. Read the user’s manual section on structured problem-solving 2. Daily schedule of applied problem- solving for practical problems
**Session 12** **Relapse prevention**	Prepare a relapse prevention plan • Strategies for encouraging generalization and maintenance	

**Figure 1:**
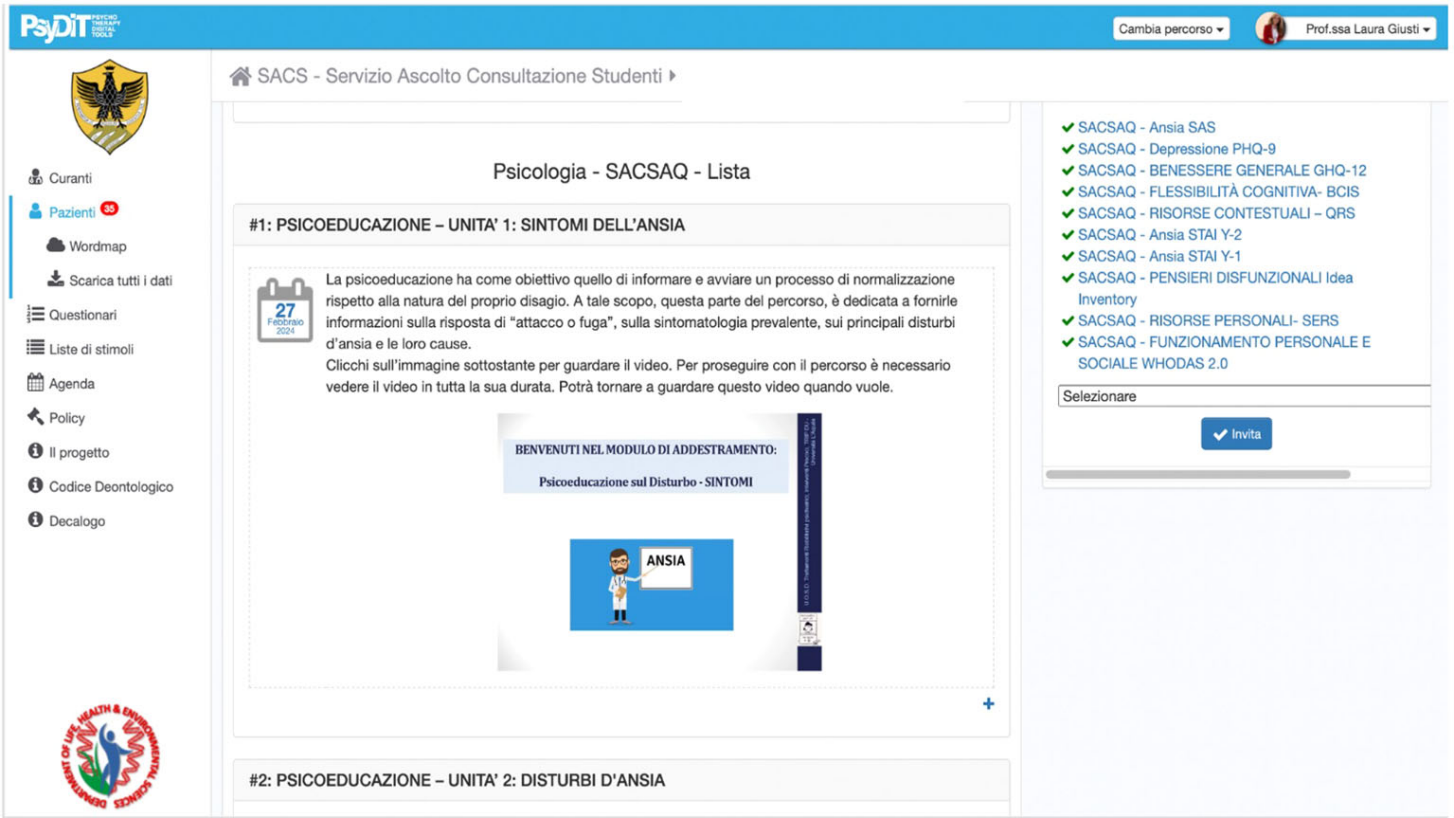
Screenshot of the Psydit.com platform with a digitized module of the CBT training for anxiety management.

Sessions were uploaded onto the digitalized platform in “slideshow” mode (.jpg format) for the theoretical part (**Figure 1**). Audio files were also uploaded and were available for download by the users. Each session lasted approximately 30 min.

The role of the therapist in the Ts-iCBT anxiety management condition was to support participant engagement with the program (specifically, checking on participant progress with modules, task reinforcement, clarification, and, where required, encouragement of reading and skill practices), to provide technological support and coping strategies for stressful events and emotional distress, primarily related to academic issues. Therapists were in a position to simultaneously help more than one user to work on their program, although these might have been at different stages of therapy.

The intervention envisaged attendance in 12 web sessions.

#### Statistical analysis

Descriptive analyses were carried out to characterize our student sample based on socio-demographic and clinical details. Continuous variables are reported as means (standard deviations), and categorical variables are reported as frequencies (percentages).

Baseline comparisons [chi-square and one-way analysis of variance (ANOVA)] were performed to assess differences between the two online intervention groups.

We developed general linear models for repeated measures analyses with a between-subjects factor (VCMH and Ts-iCBT digital interventions) and a within-subjects factor (pre-treatment–T0 vs. post-treatment–T1) for the emotional distress variable, measured by GHQ-12. All students who completed online interventions were included in the analysis. Significance was set at p < 0.05.

Statistical analyses were performed using SPSS 27.0 (SPSS Inc., Chicago, IL, USA).

## Results

### The first screening battery results

**Figure 2:**
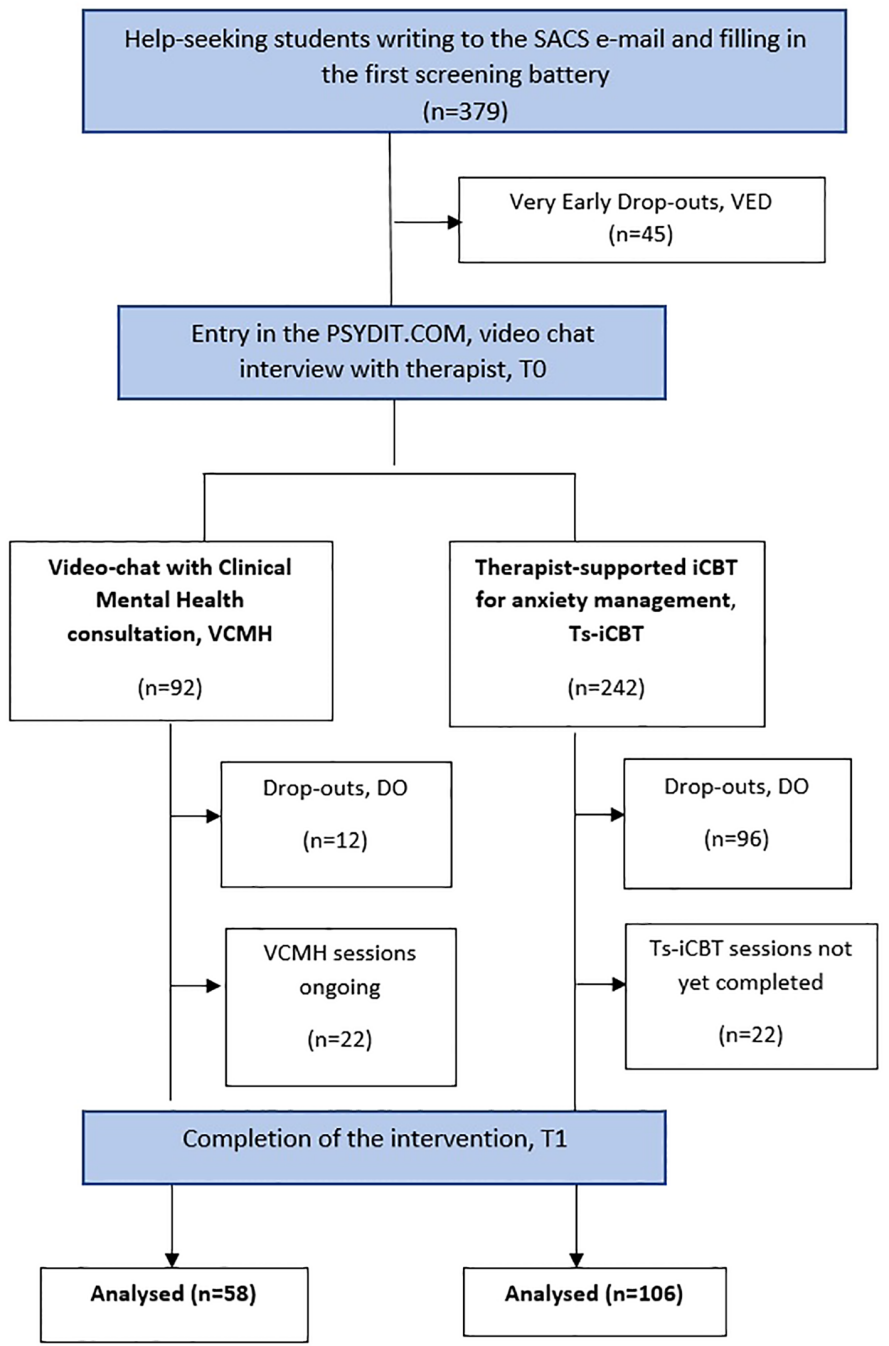
Flow of subjects through the 2-arm study in the two years.

Over the two-year period taken into account in the study, 379 students (267 women, 70.4%) contacted the service to request a consultation (**Figure 2**).

More than 10% of students who requested access to Psydit.com failed to complete registration (“very early dropouts”, VED, N = 45), with women accounting for more than half (57.8%) of this subsample; average age was 23.8 (SD = 2.9). After completing the first screening battery, students who subsequently failed to access the platform displayed statistically significant higher levels of severe traumatic distress, as measured by IES-R (IES-R = 38.5 SD = 12.3) compared to students who accessed the platform, in whom a mild psychological impact was reported (IES-R = 31.3 SD = 13.9; ANOVA: F = 10.690; p = 0.001). The former group also reported moderate depressive symptomatology, displaying statistically significantly higher mean scores at BDI-II (24.4 SD = 10.5) compared to students who accessed the platform, who displayed mild depression (BDI-II = 19.3 SD = 11.6; ANOVA: F = 7.066; p = 0.008). The former students were considered “very early dropouts”, VED.

### The online interventions results

The total of 334 subjects who accessed the platform were allocated to interventions according to their personal preferences. The majority of students seeking help chose and/or were allocated to Ts-iCBT intervention as they were not prepared to join the established waiting list. The main socio-demographical and clinical conditions of the sample are reported in **Table 2**.

**Table 2:** Main socio-demographical, living, and clinical conditions of the sample.

Variables	VCMH (N = 92)	Ts-iCBT (N = 242)
**Age (SD)**	24.1 (4.7)	23.3 (4.4)
Sex n (%)		
Women	63 (68.5)	178 (73.6)
Males	29 (31.5)	64 (26.4)
Nationality, n (%)*		
Italian	81 (88)	229 (96.6)
University courses, n (%)*		
Health professions	8 (8.6)	89 (36.8)
Medical school	18 (19.5)	37 (15.2)
Economics	4 (4.3)	9 (3.8)
Humanistic courses	8 (8.6)	18 (7.4)
Scientific courses (2 PhD students)	20 (21.7)	38 (15.8)
Psychological courses	8 (8.6)	14 (5.7)
Engineering courses	11 (11.9)	28 (11.5)
Missing	15 (16.8)	9 (3.8)
**First-year students, n (%)**	2 (15.4)	36 (20.2)
**Out-of-course students, n (%)**	4 (4.3)	34 (14)
Treated with psychopharmacological drugs by mental health services, n (%)*	14 (15.2)	14 (5.8)

*p <0.01.

No statistically significant differences were found between the two groups with regard to gender, age, out-of-course, and freshman academic conditions. Italian students seemed to display a statistically significant preference for Ts-iCBT over VCMH compared to their non-Italian counterparts (chi-square test = 8.968; d.f. = 1; p = 0.003). Students enrolled in health profession courses seemed to prefer Ts-iCBT, whereas those enrolled in scientific classes preferred to actively take part in VCMH (chi-square test = 21.562; d.f. = 6; p = 0.001).

Twenty-eight students (8.4% of total student sample, 21% of students included in interventions) were referred to mental health services and treated with psychopharmacological drugs, revealing a highly significant statistical number of subjects enrolled in VCMH compared to Ts-iCBT (chi-square test=7.722; d.f. = 1; p= 0.005).

The themes that emerged in the narrative diary were corroborated during the first interview, when professionals asked what had motivated students to seek help, with students being allowed to provide more than one reason. Professionals categorized motivations into four dimensions: academic issues, personal issues, interpersonal problems, and psychopathological problems (**Table 3**). Psychopathological problems were the most frequently reported. No statistically significant differences were detected between the three dimensions of academic, personal, and interpersonal issues in the two intervention groups.

**Table 3:** Reasons for accessing SACS reported by students.

Variables	VCMH (N = 92)	Ts-iCBT (N = 242)
Academic Issues, n (%) reported by 99 students (29.6%) in the total sample		
Difficulty in academic performance and low satisfaction	26 (28.3)	62 (26.6)
Stress related to the competitive environment	2 (2.2)	5 (2.1)
Need of re-orientation towards a more suitable course of study	1 (1.1)	3 (1.2)
Personal problems, n (%) reported by 92 students (27.5%) in the total sample		
Gender-identity issues	1 (1.1)	5 (2.1)
Feelings of inadequacy and loneliness	22 (23.9)	47 (19.4)
Autonomy needs	5 (5.4)	12 (5)
Interpersonal problems, n (%) reported by 66 students (19.8%) in the total sample		
Expectations and Family Pressure, family problems	12 (13)	30 (12.4)
Problems with friends and partners	3 (3.3)	21 (8.7)
Self-reported psychopathological problems, n (%)* reported by 138 students (41.3%) in the total sample		
Symptoms of anxiety	17 (23.3)	66 (31.3)
Depressive symptoms	18 (24.7)	17 (8.1)
Somatic symptoms	2 (2.7)	6 (2.8)
Eating disorders	4 (5.5)	1 (0.5)
Self-harm	1 (1.4)	1 (0.5)
Sleep disorders	0	3 (1.4)
Substance use	0	2 (0.9)

A statistically significant difference was found in the dimension of self-reported psychopathological symptomatology, with a different proportion of students in the VCMH group complaining of symptoms of depression and eating disorders compared to students allocated to the Ts-iCBT group, in which a higher proportion of students reported symptoms of anxiety (chi-square test = 25.148; d.f. = 7; p=0.001).

When requesting help, statistically significant differences were found, with the VCMH group presenting higher scores at GHQ-12, BDI-II, and IES-R compared to students allocated to the Ts-iCBT group (**Table 4**).

**Table 4:** Clinical measures of the two groups at entry in the study (T0).

Clinical variables	VCMH (N = 92)	Ts-iCBT (N = 242)	F	p
**GHQ-12 Total score, mean (SD)**	24.3 (7.5)	18.8 (8.8)	F = 27.597	p = 0.000
**SAS total score, mean (SD)**	54 (11.4)	51.6 (12.5)	F= 2.373	p= 0.124
**BDI total score, mean (SD)**	24.3 (10.2)	17.4 (11.5)	F = 24.474	p = 0.000
**IES-R total score, mean (SD)**	36.7 (12.9)	29.1 (13.7)	F = 20.843	p = 0.000

No statistically significant differences were found with regard to symptoms of anxiety, as measured by the SAS, in which both groups presented moderate levels of anxiety.

Throughout the two-year duration of the study, 49% of students (N = 164) out of the total sample had completed online interventions, while in 13% (N = 44) interventions were still ongoing (**Figure 2**).

In VCMH sessions, a mean number of 8 video chats were conducted (SD 6.1) with a mean once weekly therapist-led session (range 3-31 sessions; 4 students required a more extended treatment period). In Ts-iCBT, students who completed all 12 modules were committed to the intervention for approx. two-three months, with 2-3 supervised video chats with the therapist (the first after the breathing and muscle relaxation session and second - third following the cognitive restructuring and problem-solving module to verify learning of these skills).

A statistically significant higher proportion of students assigned to VCMH intervention had completed all video chats (63%) or were still continuing to attend (23.9%) with a lower rate of dropouts (13%) compared to Ts-iCBT (43.8% concluded intervention; 9.1% were still attending; 47.1% dropouts; chi-square = 36.646; d.f. 2; p = 0.000) (**Figure 2**).

Students who failed to complete online interventions (N = 126, 37,7%) were considered “dropouts”(DO) when, in the VCMH group, they had not attended two consecutive video chats and, in the Ts-iCBT group had not progressed with the planned modules for a month. Regardless of the type of intervention, therapists attempted to contact the student at least twice by e-mail and telephone through the PSYDIT.com platform.

A total of 171 VED and DO students who had contacted the SACS failed to fully exploit the opportunity of free psychological counseling support, representing a relevant 45% of the initial student sample.

On completion of the intervention in both groups, the variable emotional distress, as measured by GHQ-12, was assessed, with both forms of online interventions proving effective.

**Figure 3:**
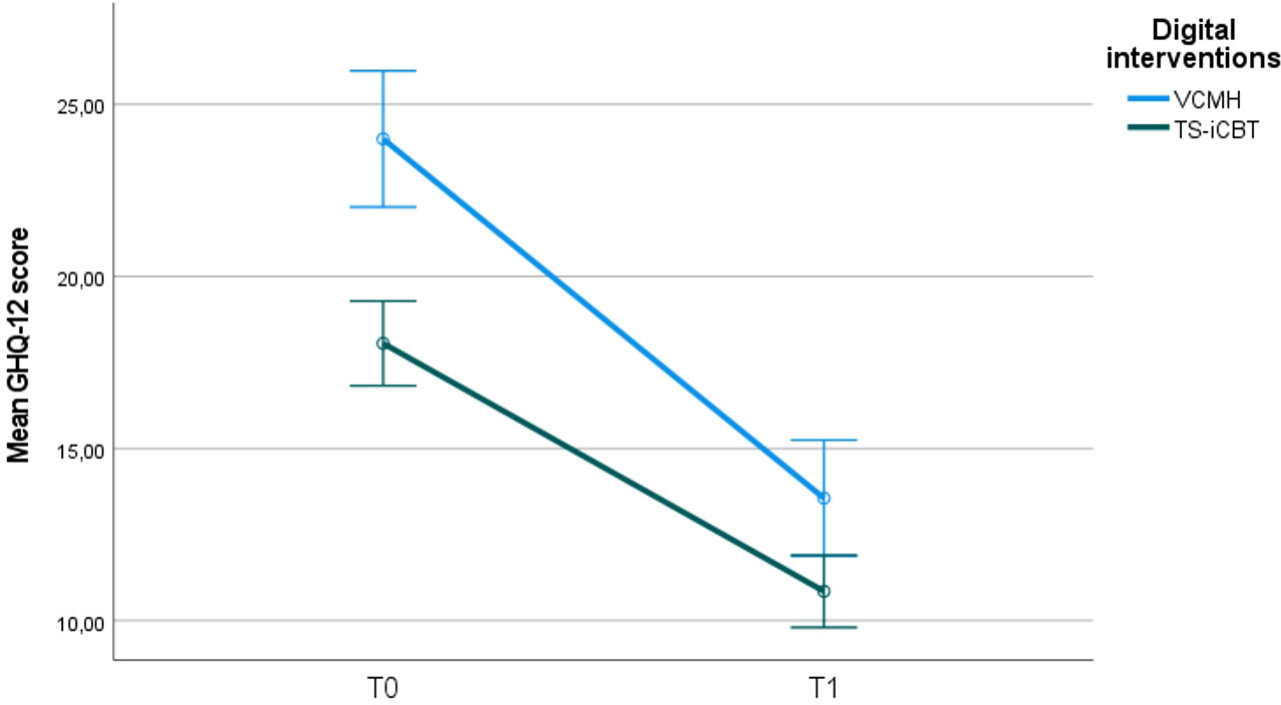
Mean change in GHQ-12 total score in the two groups at the end of intervention follow-up (T1). Error bars: 95% confidence interval.

Indeed, on completion of interventions, emotional distress, as measured by GHQ-12, displayed a statistical change over time (group for time interaction F = 1015.489; p = 0.000) in both groups and between groups (F = 5.507; p = 0.020) (**Figure 3**), revealing a better outcome for VCMH.

In addition to the above clinical variable, we investigated how many students had achieved their university degree following completion of each SACS-provided online intervention (N=47, 28.6%). In the VCMH group, 9 out of 58 students who completed intervention (15.5%) had graduated. In comparison, in the Ts-iCBT group, 38 (35.8%) out of 106 students completing intervention had achieved their academic goal, with this group accounting for a statistically significant higher percentage of graduates compared to VCMH (chi-square test: 7.782; d.f. 1; p = 0.005).

## Discussion

The findings of this study confirm the effectiveness of both forms of web intervention in managing emotional distress in students seeking help from the university counseling service. A better outcome was observed for students requesting online VCMH, who were more distressed than those opting for Ts-iCBT. Approximately one third of students attending online interventions succeeded in graduating, with a higher percentage in the Ts-iCBT group compared to VCMH. Not all students who had sought help fully exploited the opportunity of free psychological counseling, with approx. 50% of students dropping out either at a very early stage (VED), on first seeking help, or during the intervention, with a higher proportion of dropouts (DO) from the Ts-iCBT group compared to VCMH.

With regard to the effectiveness of interventions, the results obtained align with the findings of recent reports, underlining the success of counseling/psychological interventions in improving the mental health of university students. Although both face-to-face (48–50) and online interventions (22, 31, 51) are equally successful in terms of effectiveness in enhancing student well-being, following the COVID-19 pandemic, counseling services have tended increasingly towards the use of online technologies. Similar to other countries, in Italy, the COVID-19 pandemic placed an additional burden on the substantial mental strain experienced by university students (52–56), thus escalating the demand for counseling support by means of web-based online interventions. Furthermore, the pandemic also made it more difficult to access mental health services in general (57).

The cognitive-behavioral paradigm employed in our interventions seemed to be the most widely used in European psychological services for university students (58). In addition to video-chatting with therapists, the Ts-iCBT intervention was proposed based on experience gained, the limited availability of SACS resources, and the attitudes displayed by individual students, some of which experienced difficulty in openly asking for help (21). This innovative approach appeared to be highly promising and is in line with indications reported in the literature. Indeed, any additional Internet-based provisions constructed as a “semi-self-help” approach, might seem more acceptable to hesitant students compared to other web-based “face-to-face” treatments. A “go it alone” approach to managing problems may act as an effective stimulus in helping students overcome stigma relating to the use of mental health and psychological support services (59). This may indeed be one of the reasons why numerous students in the present study requested access to Ts-iCBT, displaying a preference “to handle the problem on their own” with minimal therapist support (59). Accordingly, although the two interventions provided the same behavioral and cognitive tools and strategies, Ts-iCBT requires less intervention from professionals, leaving the student a greater margin for autonomy. Furthermore, students who requested access to Ts-iCBT were found to be less distressed than those requesting online video-chat intervention.

From a clinical point of view, at the time of access to the SACS, psychopathological problems proved to be the most relevant reasons for requesting help, followed by academic, personal, and interpersonal issues. More than 40% of students presented with a range of different psychopathology symptoms (depressive symptoms, anxiety symptoms, self-harm, eating disorders, etc). During interventions, 8.4% of the sample required psychopharmacological support, with a statistically significant proportion of students in the VCMH intervention compared to Ts-iCBT. These data corroborate findings reported in the scientific literature, which underline the need to expand university counseling services, which contribute considerably toward dealing with and reducing psychopathological symptoms and distress in young students (49), and highlight the importance of addressing the mental health needs of students through fully-integrated mental health services (60).

On accessing the SACS platform, students allocated to the VCMH group were experiencing more severe symptoms of depression and emotional distress, therefore seeking support capable of addressing their multiple vulnerabilities. The different treatment choices opted for by students validate the scientific literature highlighting how more severe symptoms require an increasingly practical, feasible, and flexible treatment, which may necessitate re-evaluations and treatment modifications based on the patient’s needs (61). Moreover, NICE guidelines also suggest the use of digital interventions only in the case of mild or moderate depressive and/or anxious symptoms in young adults (62).

VCMH sessions appeared to be more effective than Ts-iCBT. The offering of psychopharmacological treatment to more severely affected students, the more flexible approach, and the longer and more tailored duration of intervention may explain these findings.

This study also investigated academic outcomes as another “hard” variable attesting to the effectiveness of interventions. Approximately 30% of students graduated, with a statistically higher proportion of graduate students in the Ts-iCBT compared to VCMH group. These data may reflect the fact that, at the time of access, students in the Ts-iCBT group did not present significant levels of emotional distress, whilst manifesting preserved global functioning. Superior executive functions such as attention, concentration, and memory, fundamental for the purpose of studying and passing exams, are affected by severe mental problems (63). Furthermore, an increase in self-esteem, influenced by feeling able to take care of one’s mental well-being independently, may positively impact not only on well-being, but also lead to an improvement in academic performance and motivation (64), although aware that many other factors (general health conditions, family environment, interpersonal relationships, socio-economic factors, 65) can influence life and academic outcomes. Furthermore, in the present study the “degree” was considered as the main, “hard” variable of academic success, which significantly reduces the possibility of evaluating more subtle dimensions of academic success, which refers to the achievement of exams, training internship, motivation to study, etc. Many of the students were freshmen or enrolled in the first years of the course, so the low percentage of graduates could be explained by these elements. Expanding these dimensions of academic success in future works would be interesting.

The finding that approx. 50% of students had dropped out of interventions was not surprising. The observed rate of 11% VED was indicative of a population affected by more severe post-traumatic and depressive symptomatology compared to students who accessed the digital platform, with the former likely experiencing impulsiveness and/or an inability to delay seeking help for an urgent “emotional need”, thus judging the level of support insufficient to address their issues. No literature comparison is available for this finding due to the fact that failing to access a platform following submission of a formal request is a “new” mode of web dropout.

In Italian university counseling services, once students have embarked on the intervention, reported dropout rates vary considerably, ranging from 4.8% at the University of Bari (66), to 20.9% at the University of Brescia (67) and 32.7% at the University of Bologna (68). All studies mentioned above refer to the pre-COVID-19 period when university counseling services provided “face-to-face intervention.” The dropout rate observed in our study was higher at 37.7%, thus more similar to the findings obtained in a study conducted at a public university in Istanbul which had already implemented online interventions prior to the COVID-19 pandemic (69). The Authors reported how more than half their sample (51.6%) dropped out of counseling sessions, with a dropout rate of 55.31% for online interventions, 38.46% face-to-face measures and 60.52% in the placebo group. In addition, another factor that may have impacted on our high drop-out rate is the “draft” presentation of the site and modules which requires further technological advances and updates to improve users’ engagement based on the students’ own suggestions and experiences.

## Strengths and limitations

To the best of our knowledge, this is the first Italian study to evaluate a large sample of students who accessed, over a two-year period (2020-2022), a university counseling service providing online interventions on a dedicated protected platform. The study included an innovative 12-module web-based asynchronous CBT intervention requiring relatively low clinical assistance. Moreover, the study took into account the most relevant “hard” academic outcomes for the specific population, i.e. obtaining a university degree.

However, the presence of four major limitations to this study should be acknowledged. Firstly, the study was not randomized; students were assigned to the two intervention arms based on their personal preferences. Secondly, the Ts-iCBT intervention was still in a “draft” form, with modules requiring further technological advancements and updates based on the suggestions and experiences of students. In a recent narrative review focused on serious games, we discussed ways in which services and therapeutic programs might facilitate access to a young, depressed population and help them maintain adherence to treatments (70). This area would need to rely on the availability of substantial technological investments, also taking into account user feedback of their experience. Thirdly, with regard to the graduation of students who completed interventions, it should be underlined how online interventions “facilitated” study and learning processes, thus likely resulting in reduced emotional distress (35) which, together with numerous other contributory factors assisted the student in reaching his or her goal. Fourth, at T1, anxiety and depression symptomatology were not evaluated with the specific standardized scales (respectively SAS and BDI), but only GHQ-12 has been re-administered, as a brief and enough comprehensive tool for emotional distress.

## Conclusions

The availability of online CBT counseling interventions represents a promising means of expanding therapeutic opportunities for university students seeking help on their academic pathway. Online services provide a growing source of suggestions on how best to personalize and tailor interventions targeting the range of diverse student needs in their “ecological living” setting, i.e. the university. The findings of the present study revealed how psychopathological symptoms are one of the main reasons for accessing university counseling services. Despite the effectiveness of online measures, students displaying a more severe symptomatology seemed to prefer therapist-led face-to-face meetings and achieved a more substantial decrease in symptoms of distress than students who opted for the digital 12-module intervention, the majority of whom presented predominantly mild symptoms of anxiety. Indeed, the latter seemed to prefer the possibility of “handling their problems on their own,” by adhering to digital online intervention in the presence of reduced therapist support, thus resulting in a reduced workload for the service. Unsurprisingly, these students reported a satisfying reduction in distress and better academic achievement.

The complex issue of dropouts constitutes a justified concern. Although web-mediated, a face-to-face therapist-led relationship appears to result in a lower rate of students discontinuing intervention. On the other hand, when left alone to pursue an asynchronous intervention, a series of factors may contribute towards student dropouts, including low flexibility, lack of engagement, or severe mental conditions. Future research should address these specific limitations by incorporating randomized controlled trials on university counselling interventions and a more detailed analysis of participant characteristics to better understand the factors influencing the effectiveness of different types of online interventions.

Indeed, university counseling services are well-suited to support traditional mental health services in the early detection and treatment of psychiatric disorders, the onset of which is typically observed in younger people (71). However, a full integration with mental health services should be deemed essential when appropriate evidence-based treatments are required by more severely affected students.

Finally, the present study is in line with the sustainable development goals proposed by the United Nations Organization, namely Goals 3 and 4. According to the United Nations (2015), Goal 3 aims to improve well-being and mental health, while goal 4 aims to provide quality, equitable and inclusive education, promoting learning opportunities, including soft skills. Well-being and education represent increasingly greater challenges and this study brings attention to those variables that can hinder the individual and professional development of young people in our country.

## Data Availability

The raw data supporting the conclusions of this article will be made available by the authors, without undue reservation.
